# The Unusual Role of Pro in Cu(II) Binding by His_2_-Cyclopentapeptide

**DOI:** 10.3390/ijms22126628

**Published:** 2021-06-21

**Authors:** Aleksandra Pieniężna, Aleksandra Kotynia, Justyna Brasuń

**Affiliations:** Department of Inorganic Chemistry, Wroclaw Medical University, Borowska 211A, 50-552 Wrocław, Poland; aleksandra.pieniezna@umed.wroc.pl

**Keywords:** cyclopeptides, coordination, copper(II) ions, potentiometric measurements, spectroscopy, proline

## Abstract

In this paper, we present findings from studying the interaction of copper(II) ions with the His_2_-cyclopentapeptide and the role of proline used for the purpose of potentiometric titration and UV-Vis, CD and EPR spectroscopic measurements. Experiments of two homodetic peptides differing by one amino acid residue were conducted for a ligand to metal ratio of 1:1 in the pH range 2.5–11.0. The presented studies reveal that peptides form only mononuclear complexes, and the CuH_2_L complex appears in the system first (for both L1 and L2). Study results show that the presence of Pro influences the structure of formed complexes and their stabilities and has a strong impact on the efficiency of copper(II) coordination.

## 1. Introduction

Proline is among the naturally occurring amino acid residues with a cyclic structure and has a significant impact on the abilities of peptides. Proline influences the peptide/protein secondary structure. The cyclic structure of the side chain causes conformational stiffness. Accordingly, the most common proline residues occur at the ends of secondary structures, such as the β-pleated sheet or α-helix [1,2]. Moreover, the Pro moiety has exceptional conformational rigidity, which reduces the flexibility of the entire molecule [3]. The double Pro-Gly motif forces antiparallel β-sheet conformation [4,5].

Of all the amino acids, proline plays a special role in the coordination of copper (II) ions by peptides. This amino acid residue can act as a “break point”, with the exception of proline present at the N-terminus. This amino acid, which is usually not directly involved in the binding of metal ions in proteins, modulates the coordination process. The presence of proline in the middle of a peptide sequence is considered an amino acid that does not directly participate in the binding of divalent metal ions. Due to the pyrrolidine ring, the nitrogen atom, which creates the peptide bond and is not able to participate in the bounding metal ions, is considered to be the “break point” of the bond cascade along the peptide chain [1].

The binding of metal ions by protein and small peptides is an important issue in the area of biochemistry and has been widely explored in recent years. Previous studies enabled the understanding of copper ion homeostasis and its role in living organisms. This trace element occurs in the prosthetic group of metalloenzymes, e.g., cytochrome c oxidase, superoxide dismutase, and tyrosinase [6,7,8]. A frequentative occurring amino acid responsible for binding divalent metal ions in protein is a side chain of His. This amino acid is often found in the active center of metalloenzymes [3].

Cyclic peptides (*CP*) have a huge potential as therapeutic agents. The specific peptide ring scaffold is resistant to enzymatic degradation, which makes them more orally bioavailable. Moreover, they are also more thermally stable than their linear analogs. Head-to-tail cyclization implies increased lipophilicity and membrane permeability [9,10]. The cyclic analogs of conotoxin (potential treatment of pain) and chlorotoxin (imaging of brain tumors) are more stable than their linear counterparts [11,12]. Cyclopeptides exhibit antimicrobial activity (*AMPs*) against Gram-positive and Gram-negative bacteria, yeasts, fungi, and viruses [13]. Some cyclopseudopeptides, such as cyclosporine A, sirolimus, and tacrolimus, are able to modulate the immune system and are used in skin disorder treatment and the prevention of organ transplant rejection [14]. The family of cyclic peptides with RGD fragments is widely studied as a radiotracer for imaging tumors [15,16,17]. This specific amino acid motif is responsible for binding to α_v_β_3_ transmembrane glycoproteins, which play a key role in tumor growth, progression, and angiogenesis [15]. This proves that the cyclization of small tetra-, penta-, and hexa- RGD peptides exhibits an increased receptor binding affinity and selectivity to integrin [18,19].

Previously, studies have shown that cyclic peptides are attractive chelators for divalent metal ions, such Hg^2+^, Pb^2+^, and Cd^2+^, and can be used as fluorescence sensors for toxic ions [20,21]. Furthermore, copper(II) complexes with *CP* could mimic antioxidant enzyme activity as a superoxide dismutase [7,22,23]. The coordination properties of *CP* could be modulated by various chemical modifications of ligands. The head-to-tail cyclization of peptide leads to the deprivation of terminal groups (NH_2^−^_ amino and –COO^−^ carboxyl), which promotes the side chains of amino acid residues for metal coordination. *CP* analogs have been shown to interact more effectively with copper(II) ions than their linear counterparts with blocked N- and C-termini [24]. Additionally, the peptide ring size of *CP* impacts the binding of Cu(II) ions. The flexibility of the peptide chain depends on the number of amino acids in the sequence, which is related to the available donor atoms for metal ions.

Accordingly, cyclopeptides in general constitute a group of compounds with an interesting topology and biology and a broad spectrum of utility. Understanding the interaction of these ligands with metal ions has high potential for application in medical and biological chemistry and underlies a detailed explanation of more complex interactions. This paper concerns basic research on the interaction of copper ions with model cyclopentapeptides and the atypical influence of the Pro residue on the interaction with metal ions.

**Scheme 1:**
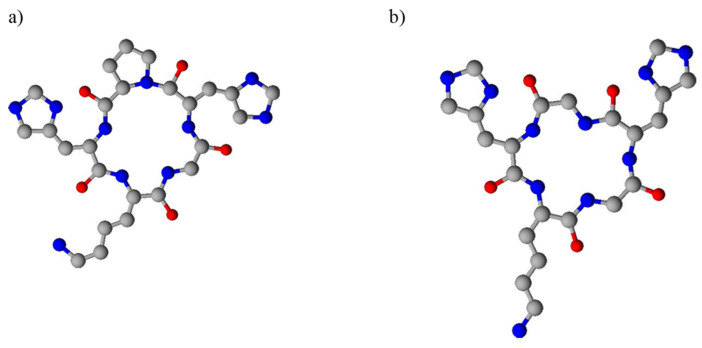
Schematic structures of investigated pentacyclopeptides: (**a**) L1—c(GlyHisProHisLys) and (**b**) L2—c(GlyHisGlyHisLys). The hydrogen atoms were omitted for simplicity; carbon atoms are grey; nitrogen atoms are blue; oxygen atoms are red.

In this work, we focused on describing the binding abilities toward Cu(II) ions of two homodetic, cyclic pentapeptides differing by one amino acid residue. The first peptide has a c(GlyHisProHisLys) sequence, whilst the second one has c(GlyHisGlyHisLys), hereinafter referred to as L1 and L2 (Scheme 1).

Owing to these facts, we decided to analyze the effect of Pro residue on the binding abilities of simple model His_2_-cyclopentapeptides. Studies were performed using an equimolar system with copper(II) ions. The application of the potentiometric titration allowed us to determine the acid/base equilibrium and stoichiometry of complexes in the studied systems, and UV-Vis, CD, and EPR spectroscopy were used to analyze atom donors directly bound to metal ions.

## 2. Results and Discussion

The acid-based abilities of the free ligands and the stability constants of formed complexes are presented in Table 1. Both ligands are characterized by three protonation constants related to the presence of two His residues and the ε-amino group on the lysine side chain and are comparable to constants found in the literature [25,26,27].

**Figure 1:**
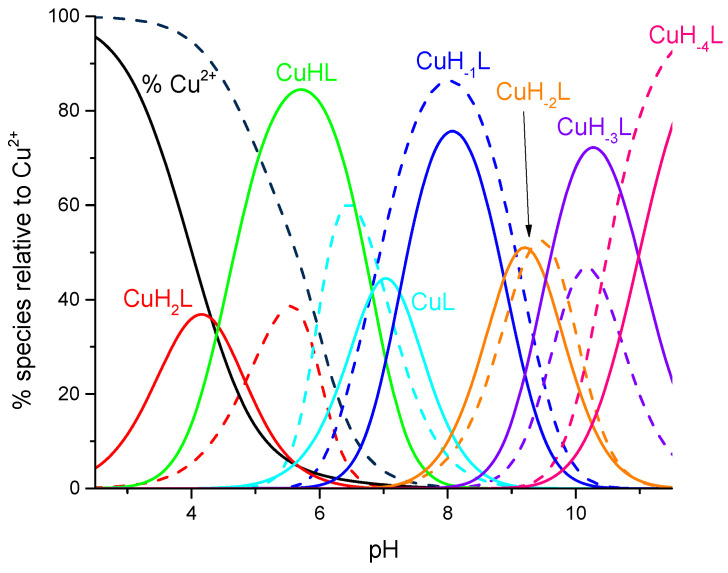
The species distribution curves for the system of L1−c(GlyHisProHisLys) (solid line) and L2−c(GlyHisGlyHisLys) (dashed line).

**Scheme 2:**
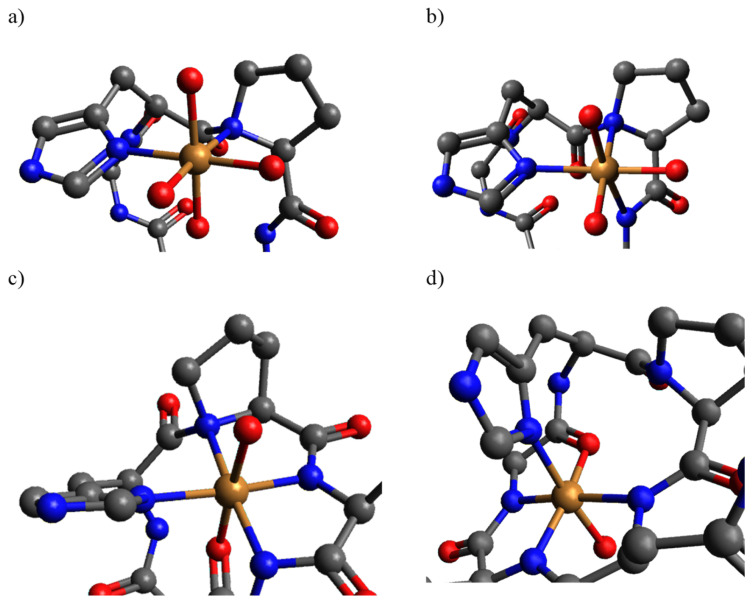
The proposed binding modes of (**a**) CuH_2_L, (**b**) CuL, (**c**) CuH_-1_L, and (**d**) CuH_-2_L visualized for L1−c(GlyHisProHisLys) peptide prepared by Avogadro Version 1.2.0.

**Figure 2:**
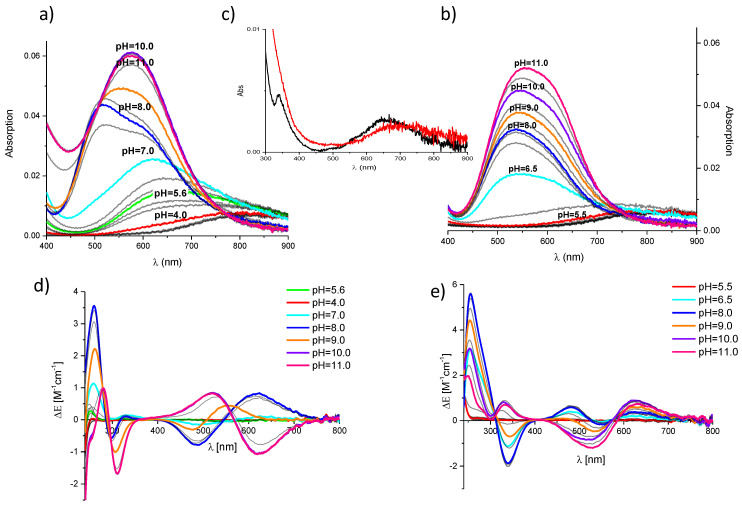
The UV-Vis spectra for the solution with an equimolar quantity of (**a**) L1 and Cu(II) ions and (**b**) L2 and Cu(II) ions, at pH between 2.5 and 11.0. (**c**) The comparison of the experimental spectra for L1 at pH 4.0 (black) and L2 at pH 5.5 (red) obtained by the subtraction of the Cu(H_2_O)_6_^2+^ spectrum. The CD spectra for the solution with an equimolar quantity of (**d**) L1−c(GlyHisProHisLys) and (**e**) L2−c(GlyHisGlyHisLys) with Cu(II) ions at pH between 2.5 and 11.0.

Generally, both peptides, L1 and L2, form complexes with the same stoichiometry (Table 1). The CuH_2_L complex appears first. Its formation is related to the dissociation of one proton from the peptide molecule, and the binding of the metal ion (Figure 1) takes place on imidazole nitrogen [28,29]. This assumption is strongly supported by the value of the corrected stability constant, logβ*_L1_ = logβ_CuH2L_ − logβ_H2L_. Its value for L2, logβ*_L2_ = 3.60, is characteristic of the complex with the {N_im_} binding mode [22,28,30,31], whilst the logβ*_L1_ for L1 is equal to 4.53 and significantly higher than that calculated for L2. The characteristic feature of L1 is the substitution of Gly by Pro residue in the HisXaaHis sequentional motif (Scheme 1). The increase in the logβ*_L1_ may be related to the extraordinary axial interaction of the metal ion with the free electron pair of amide Pro residues (Scheme 2a). This assumption is also supported by the comparison of the simulated absorption spectra, obtained by subtracting the spectrum of the copper(II) aqua ion from the spectra obtained for the discussed systems: at pH 4.0 (for L1) and 5.5 (for L2), where the CuH_2_L achieves the highest concentrations (Figure 2c). The d-d band for L1 is redshifted (λ_maxL1_ ≅ 654 nm vs. λ_maxL2_ ≅ 694 nm), although the absorption is minor due to the low concentration of the complexes, which supports additional donors in the coordination sphere of Cu(II).

In contrast to L2, with the increase in pH, L1 forms the CuHL complex. The formation of this species does not significantly influence the absorption or the CD spectra, and only an increase in the intensity of the bands was observed. This shows the same binding mode in both CuH_2_L and CuHL complexes. The value of the logK_CuH2L→CuHL_ = 6.82 is almost the same as logK_Im2_ = 6.76 in the free ligand, which suggests proton dissociation from the second imidazole ring without changes in the coordination sphere of the Cu(II) ion.

With the increase in pH, L1 and L2 form the CuL species, which is dominant around pH 7 (L1) and 6 (L2). Consequently, in the system with L1, at pH 7, there are two additional complexes CuHL and CuH_−1_L and it was not possible to obtain the spectral parameters only for the CuL species of L2. The creation of this complex by L2 is the consequence of two protons dissociating from the CuH_2_L species and the involvement of two additional N-donors in the copper(II) binding, which causes the redshift of the d-d band in the absorption spectrum (Figure 2b). Moreover, the presence of three CT bands in the CD spectrum at 253, 292, and 342 nm supports the involvement of the imidazole as well as the amide donors. In the system of L1, the creation of this species is related to the deprotonation of the amide donor and its involvement in the Cu(II) binding. Nevertheless, the comparison of the corrected stability constants for these complexes, logβ*_corr_ = logβ_CuL_ − logK_Lys_ (logβ*_L1_ = 0.03 vs. logβ*_L2_ = −1.12), shows the higher stability of the species created by L1, which may be explained by the presence of the N_Pro_ instead of the second N_Im_ in the coordination sphere of the Cu(II) ion (Scheme 1b).

Next, the CuH_-1_L form achieves the highest concentration at pH 8.0. Based on the fact that the Lys side chain is still protonated, it can be assumed that H^+^ is dissociated from the next amide nitrogen, and its coordination takes place. The UV-Vis spectrum for the L1/Cu(II) system shows a wide band with double λ_max_ = 515 and 613 nm, whilst for the L2/Cu(II) system, d-d bands at 538 nm and sh_band_ ≈ 608 nm were observed. The involvement of the second amide donor in the Cu(II) ion binding significantly influences the CD spectra recorded at pH 8.0 (Figure 2a,b). Based on the analysis presented above, the CuH_-1_L species may be described by the {(N_Im_, 2N_am_)N_Pro_} and {2N_Im_, 2N_am_} binding modes for the L1 and L2 ligands, respectively. The proposed structure of the complex when the Pro residue is located between both His moieties is presented in Scheme 2c.

Next, CuH_-2_L is only a minor species in the L1 system, but it dominates at pH 9.20 in the Cu(II)/L2 solution (Figure 1). The logK values = 8.88 (L1) and = 9.13 (L2) (Table 1) strongly support the dissociation of the proton from the subsequent peptide bond and the involvement of the next amide in the Cu(II) binding. Owing to this fact, the discussed complex may be described by the {N_Im_, 3N_am_} binding manner for L1 and L2. The spectral parameters confirm the same coordination model. Finally, the formation of the last two forms, CuH_-3_L and CuH_-4_L, has no impact on the spectral abilities of both systems, and the logK values (Table 1) support proton dissociation from the Lys side chain and water or imidazole bound to the Cu(II) ion.

**Figure 3:**
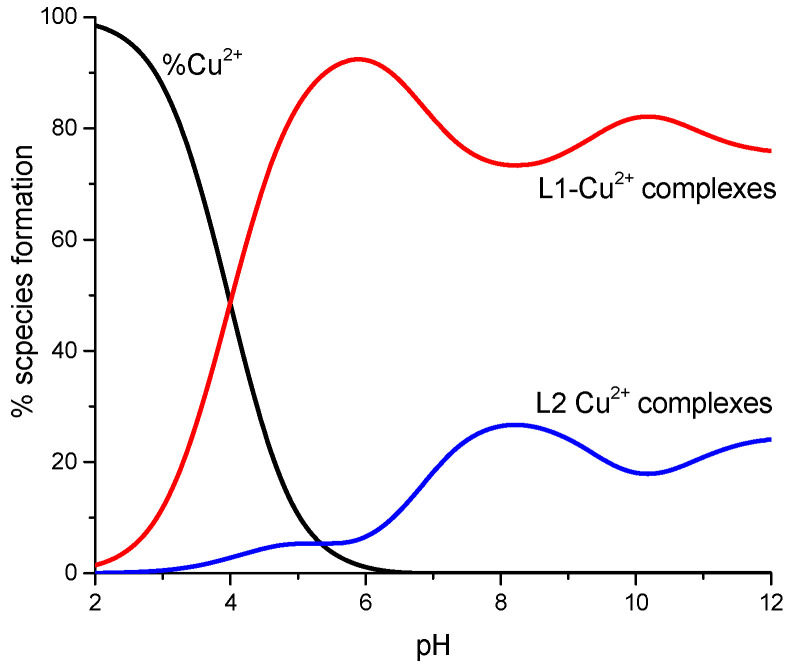
The competition plot between L1−c(GlyHisProHisLys) and L2−c(GlyHisGlyHisLys) in the presence of Cu(II) ions under the equimolar conditions.

The presence of Pro in the L1 structure influences not only the structure of the formed complexes and their stabilities, as presented above, but it also has a strong impact on the efficiency of the copper(II) coordination. Figure 3 presents the competition diagram simulated for the system L1/Cu(II)/L2 with a nL1:nCu(II):nL2 = 1:1:1 molar ratio, and it can be clearly observed that the L1 peptide binds to metal ions significantly more efficiently.

**Scheme 3:**
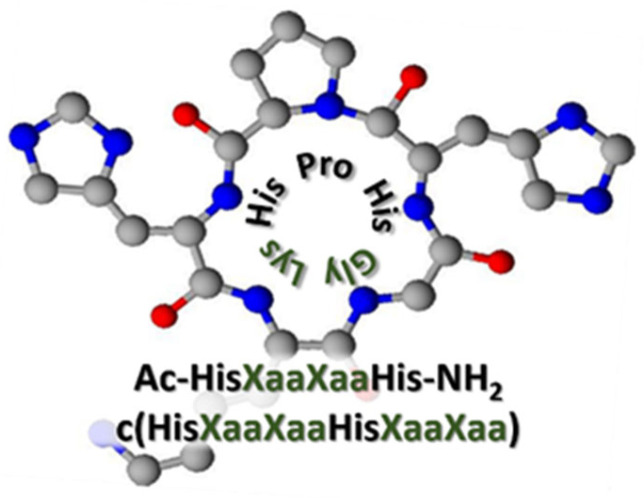
The characteristic HXXH motif presence in the investigated peptide.

The characteristic structural feature of the investigated peptide is the presence of the HXXH motif. Due to this fact, its binding abilities were compared with the properties of the simple linear and cyclic His_2_ analogs (Scheme 3).

Table 2 shows the comparison of the stability constants of the complexes with only N_Im_ in the coordination sphere of Cu(II) and with one, two, or three amide donors. It is clear that independent of the structure (linear or cyclic) of the peptide, the 1N_Im_ of the 2N_Im_ complexes of L1 are significantly more stable, which supports the postulated interaction of N_Pro_ with Cu(II) ions.

According to the durability of the complexes with amide, the presence of Pro residue in the peptide sequence promotes the coordination of the second and third amides. However, the role of Pro seems to be able to stiffen the peptide cycle, which promotes the favorable steric position of N_amide_. 

## 3. Materials and Methods

### 3.1. Materials

Both peptides, L1 and L2, were purchased commercially from STI (Poland, Poznań). The purity of the researched ligands was confirmed by the MS method. Cu(II) solution was prepared by the dilution of solid metal salts CuCl_2_ × 2H_2_O (POCH) in distilled water. For potentiometric and spectroscopic measurements, the acid solution was prepared using concentrated HCl (37%) and solid KCl to obtain the final concentration of 3.16 × 10^−3^ M and the ionic strength equal to 0.1 M. The base (KOH) was used as a titrant at 0.1 M concentration and was purchased from Sigma-Aldrich. CO_2_ was removed from the solution.

### 3.2. Potentiometric Measurements

Potentiometric studies were carried out using a Metrohm pH-metr system (Switzerland, Herisau) with a semimicro combined electrode at 25 °C calibrated in a hydrogen ion concentration using HCl [34]. The peptide concentration was 8 × 10^−4^ M, and pH-metric titration was performed at a constant ionic strength of 0.1 M KCl using sample volumes of 1.3 mL. Systems with 1:1 ligand to Cu(II) metal ion molar ratios were analyzed. Alkali (KOH) was added using a 0.250 mL micrometer syringe. Protonation constants of the ligands and stability constants (*logβ*) of the complexes and stoichiometry were calculated from titration curves using SUPERQUAD and HYPERQUAD programs [35,36].

### 3.3. Spectroscopic Measurements

The UV-Vis spectra of the copper(II) complexes were recorded on a Varian Cary 50 Bio spectrophotometer (Varian Inc., USA, Palo Alto, California) in 1 cm path length quartz cells. All UV-Vis spectra were collected in the 350–900 nm and 2.5–11 pH range. Circular dichroism (CD) spectra were recorded on a Jasco J-1500 spectrophotometer (Jasco, Japan, Tokyo) in the 240–800 nm range using 1 cm cuvettes. The same concentration, ionic strength, and molar ratio were used for both spectroscopic and potentiometric studies. 

## 4. Conclusions

The coordination abilities and impact of proline on the two cyclopentapeptides c(GlyHisProHisLys) and c(GlyHisGlyHisLys) are presented in this paper. Studies were conducted on Cu(II) ions with a ligand-to-metal ratio of 1:1. Both peptides form complexes with the same stoichiometry. The coordination started with the CuH_2_L complex. At pH 6–7, CuL is the dominant species. The final solution of both ligands contains species with the {N_Im_, 3N_am_} binding mode.

The competition between L1 and L2 in the presence of Cu(II) ions under equimolar conditions shows that the L1 peptide with proline in the sequence binds to metal ions significantly more efficiently. Additionally, complexes of L1 are significantly more stable, which strongly supports the extraordinary axial interaction of N_Pro_ with Cu(II) ions.

## Figures and Tables

**Table 1:** The protonation constants and stability constants for ligands L1—c(GlyHisProHisLys) and L2—c(GlyHisGlyHisLys) calculated by fitting potentiometric curves by HYPERQUAD.

	L1		L2	
	*logβ*	*logK*	*logβ*	*logK*
**HL**	9.79 ± 0.04	9.79	9.63 ± 0.05	9.63
**H_2_L**	16.53 ± 0.06	6.74	16.71 ± 0.08	7.08
**H_3_L**	21.89 ± 0.06	5.36	22.53 ± 0.08	5.82
				
**CuH_2_L**	21.06 ± 0.05	4.42	20.31 ± 0.07	−
**CuHL**	16.64 ± 0.03	6.82	−	−
**CuL**	9.82 ± 0.05	7.25	8.51 ± 0.06	6.90
**CuH_-1_L**	2.57 ± 0.04	8.88	1.61 ± 0.05	9.13
**CuH_-2_L**	−6.31 ± 0.04	9.53	−7.52 ± 0.07	9.87
**CuH_-3_L**	−15.84 ± 0.05	10.98	−17.39 ± 0.07	10.40
**CuH_-4_L**	−26.82 ± 0.05	−	−27.79 ± 0.07	−

**Table 2:** Calculated value of logβ* independent of the protonation state of the selected ligands.

*logβ* Independent of the Protonation State of the Ligand					
Ligand	1N_Im_	2N_Im_	1N_am_	2N_am_	3N_am_
**Ac-HGGH-NH_2_** [32]	−	5.97	−	−7.88	−16.10
**Ac-HAAH-NH_2_** [32]	−	6.08	−0.93	−8.05	−16.39
**Ac-HVVH-NH_2_** [33]	−	5.79	−1.05	−9.29	−17.60
**c(HGKHP)—L1**	4.54	6.85	0.03	−7.22	−15.84
**c(HGGHGG)** [22]	3.63	5.45	−1.40	−8.00	−
logβ* = **log*β_CuHxL_ −* log*β_HxL_***					

## Data Availability

Not applicable.
